# Dissimilatory Nitrate Reduction to Ammonium in the Yellow River Estuary: Rates, Abundance, and Community Diversity

**DOI:** 10.1038/s41598-017-06404-8

**Published:** 2017-07-28

**Authors:** Cuina Bu, Yu Wang, Chenghao Ge, Hafiz Adeel Ahmad, Baoyu Gao, Shou-Qing Ni

**Affiliations:** Shandong Provincial Key Laboratory of Water Pollution Control and Resource Reuse, School of Environmental Science and Engineering, Shandong University, Jinan, 250100 China

## Abstract

Dissimilatory nitrate reduction to ammonium (DNRA) is an important nitrate reduction process in estuarine sediments. This study reports the first investigation of DNRA in the Yellow River Estuary located in Eastern Shandong, China. Saltwater intrusion could affect the physicochemical characteristics and change the microbial community structure of sediments. In this study, the activity, abundance and community diversity of DNRA bacteria were investigated during saltwater intrusion. The slurry incubation experiments combined with isotope-tracing techniques and qPCR results showed that DNRA rates and *nrfA* (the functional gene of DNRA bacteria) gene abundance varied over wide ranges across different sites. DNRA rates had a positive and significant correlation with sediment organic content and extractable NH_4_^+^, while DNRA rates were weakly correlated with *nrfA* gene abundance. In comparison, the activities and abundance of DNRA bacteria did not change with a trend along salinity gradient. Pyrosequencing analysis of *nrfA* gene indicated that *delta-proteobacteria* was the most abundant at all sites, while *epsilon-proteobacteria* was hardly found. This study reveals that variability in the activities and community structure of DNRA bacteria is largely driven by changes in environmental factors and provides new insights into the characteristics of DNRA communities in estuarine ecosystems.

## Introduction

Rapid economic development and human activities are the major source of nutrients such as nitrogen which, when released into rivers, can cause the problem of eutrophication. Moreover, estuaries are affected by runoff and ocean power dynamics interactions, where many nitrogen cycles coexist^1^. Estuarine sediment is not only the source of nitrogen but also the sink of nitrogen, which is deserving of study.

Denitrification, dissimilatory nitrate reduction to ammonium (DNRA) and anaerobic ammonium oxidation (anammox) are the key processes related to nitrate reduction. It is thought that denitrification and anammox are the main pathways for nitrogen removal in estuaries and that 50% of the N load can be removed as N_2_ or N_2_O by denitrification^2^. However, DNRA converts nitrate to ammonium via nitrite and conserves nitrogen in estuaries. In recent years, it is reported that nitrogen can be removed due to anammox coupled with dissimilatory nitrate reduction to ammonium^3^. DNRA exists in various aquatic systems including estuaries, which plays an important role in sediments^4–7^. The distribution of DNRA bacteria in estuaries is an important factor affecting water pollution.

DNRA and denitrification compete for NO_3_^−^ under hypoxic or anaerobic conditions. The environmental factors control this competition including labile organic carbon, nitrate availability, the ratio of electron donor/acceptor (carbon/nitrate), sulfide concentration, soil sand content, pH, microbial generation time (the growth rate of the cultivated bacteria in a chemostat), NO_3_^−^/NO_2_^−^ and temperature^8–13^. The importance of DNRA in estuarine sediments depends on genetic and biogeochemical investigation. Lots of studies have shown that DNRA dominates in electron donor-rich zones with a low nitrate availability and higher temperature^14, 15^. The DNRA process can be divided into two steps^16, 17^. The first step is nitrate reduction to nitrite, such as denitrification, which is catalysed by periplasmic or membrane-bound nitrate reductases (NARs). *napA* and *narG* are genes coding these NARs, respectively. Following nitrate reduction, nitrite is reduced to ammonium through nitrite reductase (NIR), encoded by *nrfA* gene. In comparison, *nirS* and *nirK* are related nitrite reductase genes involved in denitrification, while *nosZ* encodes nitrous oxide reductase. Through the quantification of these functional genes, the proportion of DNRA bacteria in nitrate reduction is present. In addition, DNRA bacteria are diverse. Except fermentative bacteria, some sulfur bacteria and some anammox bacteria can be disguised as DNRA bacteria^18–21^.

Currently, there have been many estuarine studies on DNRA conducted in Europe and America^22–25^, while in case of China, DNRA has mainly been studied in the Pearl River estuary^26–28^. The effect of carbon source and the rate of nitrate reduction were examined in these studies. Deng *et al*. estimated the contributions of anammox, denitrification, DNRA on nitrate reduction in the Yangtze Estuary^29^. However, the abundance and community composition were not explored based on *nrfA* gene analysis. The Yellow River is the second longest river in China, and it flows into the Bohai Sea. The Yellow River estuary has high nitrogen loading due to anthropogenic and agricultural activities. In addition, intrusion of saline water may support DNRA^30^. Another study has shown that higher salinity is associated with high rates of DNRA, while lower salinity favours high rates of denitrification^31^. Therefore, we examined the activity and community structure of DNRA bacteria from the estuary mouth to the flying bridge when the saltwater intruded into freshwater. The aims of this study were (1) to investigate the activity and the abundance of DNRA bacteria along the Yellow River Estuary and their correlation, (2) to explore the composition of DNRA community based on pyrosequencing techniques and to (3) evaluate the relationships among important environmental factors and the distribution of DNRA bacteria. For this study, DNRA rates were measured via a slurry incubation experiment combined with an isotope-tracing technique, while the abundance was quantified using quantitative real time polymerase chain reaction (qPCR) of *nrfA* gene, which has been perceived as the functional gene of DNRA bacteria. High-throughput sequencing of *nrfA* gene was conducted to explore the diversity of DNRA community.

## Results

### Environmental parameters

Bottom water and sediment parameters of the five sites are listed in Supplementary Table S1. Bottom water NO_3_^−^ and NH_4_^+^ levels were extremely high due to extensive anthropogenic and agricultural activities. It was found that the concentrations of NO_3_^−^ decreased gradually along the estuary, whereas the salinity increased from 4.2 to 22.1‰. The elevated salinity was caused by saltwater intrusion. Over this same area, the colour of the water in the Yellow River Estuary changed from yellow to blue. Bottom water was alkaline, and the pH value varied from 7.94 to 8.08. The dissolved oxygen (DO) in bottom water varied slightly among the sites, ranging from 8.92 to 10.31, which exceed air equilibration (Supplementary Table S2). Sediment organic carbon (SOC) and extractable NH_4_^+^ were much higher at sites 3 and 5, especially at the flying bridge site 5, where sediment organic carbon and extractable NH_4_^+^ reached 5.37 g/kg and 0.81 μmol g^−1^, respectively, which are nearly two times higher than those at site 3. Correspondingly, lower sand content and greater moisture content occurred at these two sites.

### DNRA rate and *nrfA* gene abundance

**Figure 1:**
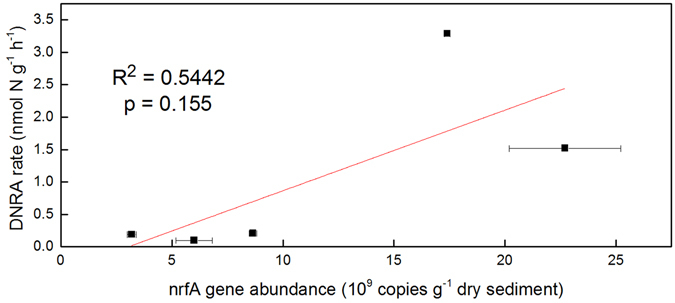
Correlations between DNRA rate and *nrfA* gene abundance. The linear regression is shown for the relation between DNRA activities and abundance. R^2^ represents regression coefficient while p represents regression significance. Error bars represent standard deviation.

The potential rates and the abundances of DNRA bacteria based on *nrfA* gene quantification in the sediments of sampling sites are shown in Supplementary Table S3. DNRA rates varied significantly among five sites, ranging from 0.10 to 3.29 nmoles N g^−1^ h^−1^. The highest DNRA rate was found at site 5, which is located around a flying bridge, while lower rates were measured in the sediment samples of sites 1, 2 and 4. Higher transcripts of *nrfA* gene were found at sites 3 and 5, with values of 2.27 × 10^10^ and 1.74 × 10^10^ copies g^−1^ dry sediment, respectively. The activities of DNRA bacteria are not significantly correlated with *nrfA* copies (Fig. 1).

### Diversity of DNRA community

Via high-throughput sequencing, raw sequences were obtained from the sediment samples of Yellow River Estuary. After the removal of low quality reads, a total of 114,138–205,444 valid sequences were produced. Then, a total of 104,395–174,960 high quality sequences for these samples were obtained via further filtering, which were clustered into operational taxonomic units (OTUs) ranging from 4,511 to 6,093 (Supplementary Table S4). The relationships among the OTUs of these four samples are illustrated in Supplementary Fig. S1. As shown in this figure, site 3 and site 5 shared the highest number of OTUs than any other two sites, while site 1 shared few OTUs with other sites. Alpha diversity indices including ACE, Chao1, Simpson and Shannon are listed in Supplementary Table S4. The ACE and Chao 1 estimators showed that the highest community richness occurred at site 5, while the lowest richness occurred in the estuary mouth site 1. Both the Simpson index and the Shannon index of the sediment samples of sites 5 and 1 were higher than those of the other two sites, which indicated that higher diversity was found in these two sites, though the difference was not substantial. Similarly, the same trend of sequence evenness with community richness was found (Fig. S2). The line representing site 5 was the smoothest, and site 5 had the highest community evenness.

**Figure 2:**
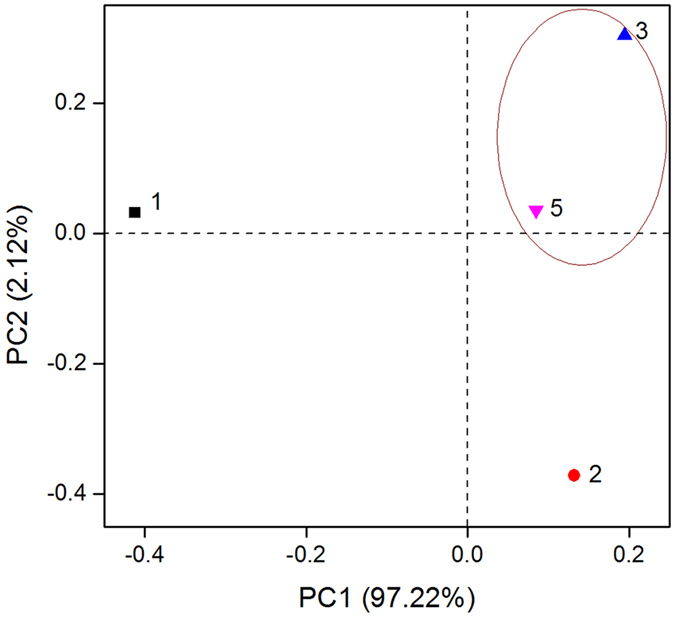
PCA analysis of DNRA communities in the Yellow River Estuary. The circle indicates that the communities in the two sites share higher similarities. Percentages represent the amount variance explained by each dimension.

To compare the DNRA communities of four sites, principal components analysis (PCA) based on community composition at genus level was performed^32^. Figure 2 shows the spatial variation of four samples with 97.22% (PC1) and 2.12% (PC2) of the variance explained. It indicated that site 3 and site 5 were clustered together, and the community structures of these two sites were similar. The community composition of site 2 also shared some similarities with those of sites 3 and 5. However, the community of site 1 was the most distinct of all the samples. This phenomenon is demonstrated clearly in the Venn diagram (Supplementary Fig. S1).

**Figure 3:**
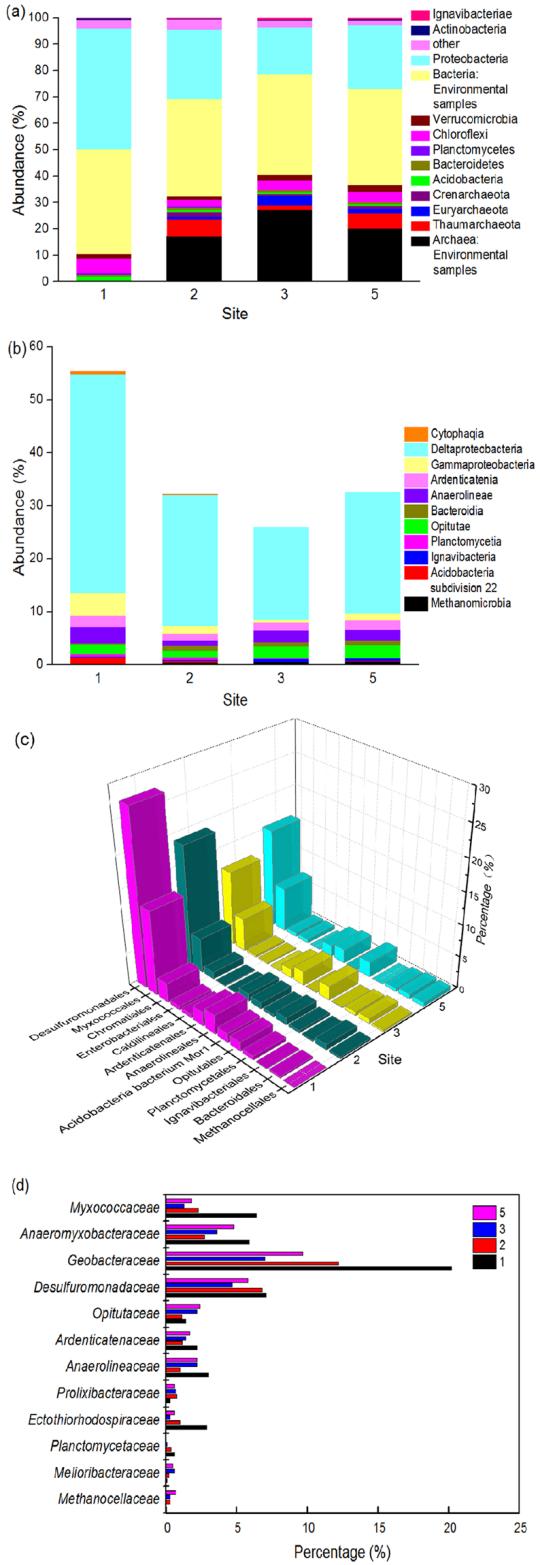
Distributions of DNRA bacteria in the Yellow River Estuary at different levels. (**a**) The phylum level; (**b**) the class level; (**c**) the order level; (**d**) the family level.

After removing rare OTUs, modified OTUs were classified into different levels such as phylum, class, order and so on. The classification results are listed in Supplementary Table S5. In this study, a total of 20 bacterial phyla, 3 archaeal phyla, and 3 eukaryotic phyla across all samples were identified. Dominant phyla (>0.5% sequence abundance in at least one site) are listed in Fig. 3a. In addition, many sequences based on *nrfA* pyrosequencing were not classified to certain type, and they appeared in sediment samples from home and abroad. For archaea, *Thaumarchaeota* and *Euryarchaeota* were the two predominant phyla, accounting for 0–6.3% and 0–3.5%, respectively. However, no archaea were observed in site 1. *Proteobacteria* was the most abundant phylum in all samples, accounting for 18.0–45.9%. Other dominant phyla were *Chloroflexi* (2.4–5.5%), *Verrucomicrobia* (1.3–2.4%), *Acidobacteria* (0.5–1.9%) and *Bacteroidetes* (0.8–1.0%). In addition, small quantities of *Planctomycetes* and *Actinobacteria* were also present.

At the class level, as shown in Supplementary Table S5, a total of 47–58 classes in four sites were obtained, and 10 of them were relatively abundant (>0.5% sequence abundance in at least one site). *Deltaproteobacteria* was the most abundant of all sites, accounting for 17.3–41.2% (Fig. 3b). *Gammaproteobacteria* was the second most abundant in *Proteobacteria* with abundance of 0.7–4.3%, and other *Proteobacteria* hardly existed. *Methanomicrobia* was the dominant class of archaea, accounting for 0–0.7%. The other main bacterial classes were *Anaerolineae*, *Ardenticatenia* and *Opitutae*. The proportion of *Acidobacteria subdivision 22* in site 1 was higher than that in the other three sites (Fig. 3b).

In this study, a total of 55–68 orders among four sites were detected, and 13 of them were predominant (>0.5% sequence abundance in at least one site). The 13 orders accounted for 25.2–53.8% of all sequences. As depicted in Fig. 3c, *Desulfuromonadales* (11.8–27.4%) was the most abundant order, and *Myxococcales* (5.2–13.2%) was the second most abundant. They both belong to *Deltaproteobacteria*. The proportion of *Anaerolineales* and *Ardenticatenales* exceed 1.0% in the order of site 1 > site 5 > site 3 > site 2. *Chromatiales* (*Gammaproteobacteria*) was slightly more abundant than other orders, accounting for 0.3–3.0%, especially in the site 1 (3.0%). Among the 13 orders, other relatively abundant orders including *Opitutales* (1.1–2.4%), *Enterobacteriales* (0.1–0.6%), *Acidobacteria bacterium Mor1* (0.1–1.4%), *Ignavibacteriales* (0.1–0.6%), and *Bacteroidales* (0.3–0.9%) were shared by all four estuary sites. *Cytophagales* (*Bacteroidetes*) was only shared by site 1 and site 2 at a lower level (0.6% and 0.1%, respectively).

Of all filtered sequences, a total of 72–86 families were detected (Supplementary Table S5). The 12 dominant families are listed in Fig. 3d. *Geobacteraceae*, *Desulfuromonadacea*, *Anaeromyxobacteraceae* and *Myxococcaceae*, which are members of *Deltaproteobacteria*, were more abundant than other families, accounting for 16.8–40.2% of the classified sequences. Each of these four families, plus *Anaerolineaceae*, *Ardenticatenaceae* and *Ectothiorhodospiraceae* in site 1, was the most abundant in all sites. Other abundant families were *Methanocellaceae*, *Opitutaceae*, *Melioribacteraceae*, *Planctomycetaceae*, *Prolixibacteraceae* and *Flammeovirgaceae*. Among them, *Planctomycetaceae* was present in three of the sites at low levels, though not at site 5. *Methanocellaceae* was present in site 2, site 3 and site 5, while no archaea were present in site 1.

**Figure 4:**
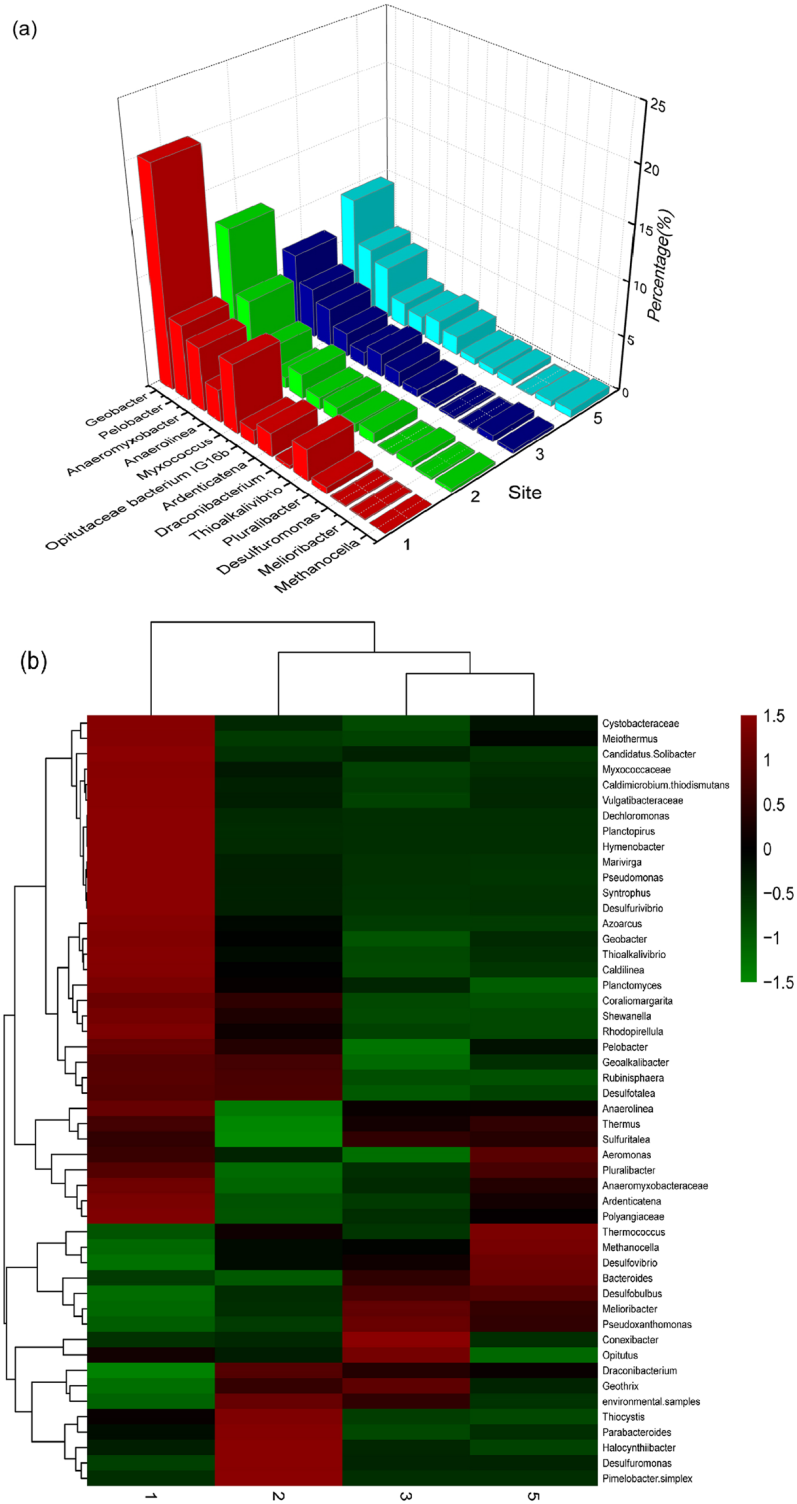
Taxonomic classification of sequences from DNRA bacterial communities in the Yellow River Estuary. (**a**) The genus level; (**b**) richness heat map of the 50 most abundant genera.

A total of 95–114 genera were identified in the four sites, including 13 predominant genera (>0.5% abundance in at least one site). As shown in Fig. 4a, *Geobacter* was the most dominant genus at all sites, accounting for 7.0–20.2%. However, the proportion of *Geobacter* in site 1 was about two or three times that of other sites. *Pelobacter*, *Anaeromyxobacter* and *Myxococcus* were more abundant than other genera, and altogether they accounted for 9.5–19.3% of the classified sequences. These four genera belong to *Deltaproteobacteria*. In addition, some genera that belong to *Gammaproteobacteria* were also present. *Thioalkalivibrio* was the most abundant in site 1, accounting for 2.9%. *Pluralibacter* was present at lower levels in every site, accounting for 0.1–0.6%. *Desulfovibrio* (*Deltaproteobacteria*) and *Shewanella* (*Gammaproteobacteria*) were present at a very low level in three sites (<0.3%). Other abundant genera were *Desulfuromonas* (0.1–0.5%), *Ardenticatena* (1.2–2.2%), *Anaerolinea* (1.0–3.0%), *Methanocella* (0–0.7%), *Draconibacterium* (0.3–0.8%), *Melioribacter* (0.1–0.6%) and *Opitutaceae bacterium IG16b* (0.1–0.6%). The 50 most abundant genera were selected to create a heatmap using cluster analysis (Fig. 4b). It was obvious that the community structure was more similar between site 3 and site 5. Then, they were clustered into a group with site 2. The 33 upper genera were more abundant in the site 1, and their colour was red. In the same way, the eight genera at the bottom of the figure were more abundant in site 2 (Fig. 4b).

### Relationships of DNRA rates, abundance, diversity and environmental parameters

**Figure 5:**
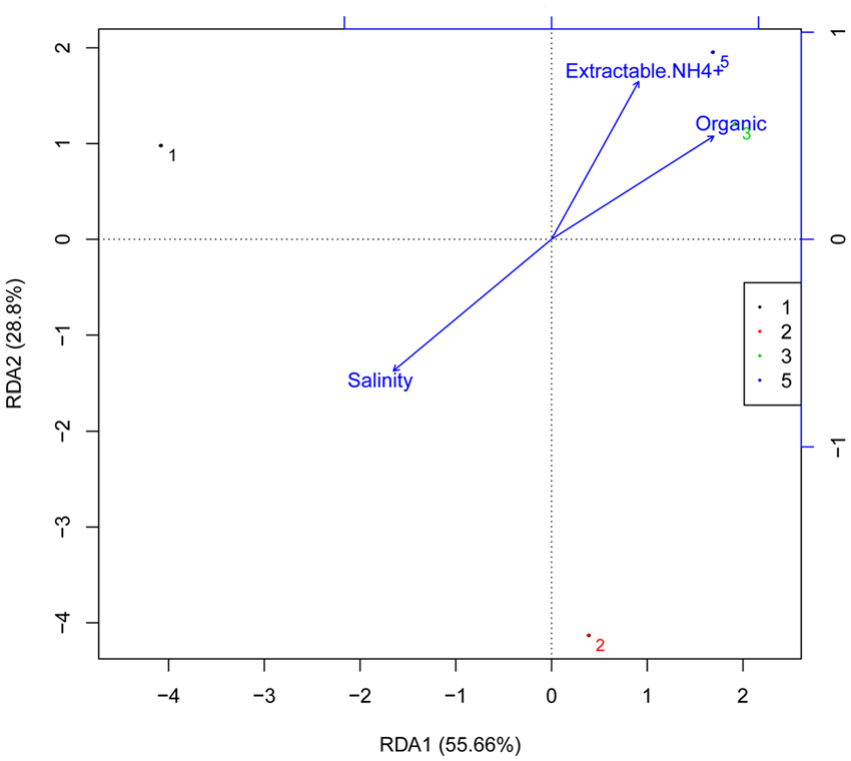
Redundancy analysis (RDA) plot to investigate the correlation between the DNRA bacteria distribution and dominant environmental parameters.

Pearson’s correlation analysis was conducted to examine the relationship between the distribution of DNRA community and environmental variables. Extractable NH_4_^+^, organic carbon and moisture content of sediments were significantly and positively correlated with DNRA rates (P_NH4_ = 0.005, P_organic_ = 0.004, P_moisture_ = 0.004), while the correlation between these three characteristics and *nrfA* gene abundance was not significant (P_NH4_ = 0.258, P_organic_ = 0.103, P_moisture_ = 0.259) (Table 1). *nrfA* gene copy number had a positive and significant correlation with pH in bottom water (Table 1). However, it was unexpected that DNRA rates and *nrfA* gene abundance had no positive and significant relationship with salinity. The correlation coefficients are −0.722 and −0.638, respectively (Table 1). Redundancy analysis (RDA) analysis confirmed the Pearson’s correlation results (Fig. 5), showing that salinity had a weak effect on community structure of these four sites, and other environmental factors had no obvious effects on the activity and abundance of DNRA bacteria. Regarding the diversity of the DNRA community, the Chao1 index, which represents richness of *nrfA* sequences, had a positive and significant correlation with the concentration of nitrate in the bottom water.

**Table 1:** Pearson’s correlation coefficients of DNRA rate, *nrfA* gene abundance, diversity index and environmental parameters (n = 5 for *nrfA* gene abundance and DNRA rate; n = 4 for Chao1 and Shannon).

	Salinity	pH	DO	NH_4_^+^	NO_3_^−^	Moisture content	Organic carbon	Extractable NH_4_^+^
**Coefficients**								
*nrfA* gene abundance	−0.722	**0.914**	0.872	−0.612	0.657	0.626	0.802	0.627
DNRA rate	−0.638	0.654	0.459	−0.283	0.588	**0.977**	**0.979**	**0.974**
Chao1	−0.891	0.841	0.845	−0.905	**0.974**	0.782	0.907	0.629
Shannon	−0.255	0.082	−0.189	0.549	0.084	0.455	0.285	0.680
***P*** **values**								
*nrfA* gene abundance	0.169	0.030	0.054	0.273	0.228	0.259	0.103	0.258
DNRA rate	0.246	0.232	0.437	0.644	0.297	0.004	0.004	0.005
Chao1	0.109	0.159	0.155	0.095	0.026	0.218	0.093	0.371
Shannon	0.745	0.918	0.811	0.451	0.916	0.545	0.715	0.320

## Discussion

In recent years, due to rapid economic growth, agricultural development and industrialization, large quantities of untreated wastewater have been discharged directly into the Yellow River, causing a high nitrogen loading with nitrate and ammonium concentrations of 102.86–272.14 μM and 110.71–155.71 μM, respectively. The nitrate concentration decreased along the Yellow River Estuary due to saltwater intrusion, though all values far exceed those of many estuaries abroad^24, 33^. However, there was little nitrate in the sediment. DNRA rates are comparable to some previous studies in some aquatic systems. Table 2 lists DNRA rates and corresponding contributions to nitrate reduction in different biological environments (e.g., river, mangrove, estuary, marine, and paddy) around the world. In this study, DNRA rates have a wide range of 0.10–3.29 nmol N g^−1^ h^−1^, which indicated that the activities of DNRA bacteria varied between different sites in the Yellow River Estuary. Because the environmental parameters of sites in this study were similar with those in some sites of the Yangtze Estuary, the activities of denitrification and anammox could be used to estimate the contribution of DNRA to total nitrate loss. According to Table 2, DNRA contributed 8.68–42.07% of the nitrate reduction.

**Table 2:** DNRA rates and corresponding contributions to nitrate reduction in various ecosystems.

Locations	DNRA rate (nmol N g^−1^ h^−1^)	DNRA (%)	References
North Atlantic Continental Shelf	0.83–4.17	<0.2	58
River Cole Floodplain	0.01–0.71	8 (7–10)^a^	48
Tuvem Mangrove	27.1–49.6	≈99	59
Colne River Estuary	2.2–25.1	59 (44–74)	33
Cape Fear River Estuary	0–1.89	2.56 (1.9–44.6)	23
Yangtze Estuary	0.03–0.89	25.8 (3–45)	29
Shanghai urban river networks	0–10.3	9 (0–50)	4
Santa Fe River Tributaries	3.13–11.67	NA^b^	60
Typical Chinese Paddy Soils	0.03–0.54	0.54–17.63	7
Yellow River Estuary	0.10–3.29	8.68–42.07^c^	This study

^a^The percentage of DNRA in the total NO_3_^−^ reduction (denitrification + DNRA); the rest studies in the column show the percentage of DNRA in the NO_3_^−^ reduction (denitrification + DNRA + anammox). ^b^NA represents no available data. ^c^The percentage of DNRA in the total nitrate reduction were estimated where rates of denitrification and anammox in some sites of the Yangtze Estuary were used to serve as data in this study.

The higher DNRA rates occurred at site 3 and site 5, where the salinity ﻿values﻿ were lower while the extractable NH_4_^+^ levels and organic carbon contents were higher. In this study, extractable NH_4_^+^ and organic carbon content were more important than salinity. DNRA prefer a low NO_3_^−^ and high labile organic carbon environment^34^. If the salinity of site 3 and site 5 were to be elevated, DNRA rates may greatly increase. Correspondingly, there were higher *nrfA* gene abundances in the sediments of site 3 and site 5. The abundances of DNRA bacteria in the Yellow River Estuary were higher than those in other estuaries^23, 33, 35^, but the abundances were an order of magnitude lower than the amount of denitrifying bacteria (Supplementary Fig. S3). Those abundances were also positively correlated to pH. Some studies have shown that more alkaline conditions favoured DNRA^36, 37^.

Community diversity was another important and relevant element of DNRA. Because of the similar environmental parameters, including high organic content and high extractable NH_4_^+^ levels at site 3 and site 5, the community structures of these two sites were very similar. However, site 1 was the junction of the Yellow River and the Bohai Sea, and more uncultured bacteria existed here, which caused the bacterial composition to be much more distinctive. This unique distribution contributed to higher community diversity of site 1 following site 5. Site 5 was located under the flying bridge and was the most polluted site due to more human activities. Therefore, DNRA was elevated in polluted systems. Similar conclusions have been reached in many studies^28, 38, 39^. In this study, 11 dominant phyla could perform DNRA, including three archaeal phyla. *Thaumarchaeota, Euryarchaeota* and *Crenarchaeota* could perform DNRA in a proportion of 6.1–9.0%, except at site 1. However, no *nrfA* sequences were encoded in the available archaeal genomes in a previous study^40^. In addition, the authors found that *NrfA* proteins of the main clades are populated with taxa from six different phyla (i.e., *Proteobacteria*, *Verrucomicrobia*, *Acidobacteria*, *Planctomycetes*, *Firmicutes* and *Chloroflexi*) through the phylogenetic analysis based on *nrfAF2aw* and *nrfAR1* primers. *Bacteroidetes* and *Acidobacteria* were also members of DNRA bacteria^5, 41^. The results were consistent with our research with the exception of *Firmicutes*. At class level, DNRA bacteria could be classified into four clusters according to previous literature^35, 38^. delta-proteobacteria (I), epsilon-proteobacteria (II), gamma-proteobacteria (III) and the last cluster were *Chlorobium phaeobacteroides* and bacteria from anammox reactor^41, 42^. As shown in Fig. 3b, no *epsilon-proteobacteria* was found in the Yellow River Estuary, and *delta-proteobacteria* was the most abundant. Among *delta-proteobacteria*, various members of the *Desulfomonadales* and *Myxococcales* are capable of anaerobic respiration utilizing organic compounds as electron donors. At the same time, *Geobacter*, which was assigned to *Desulfomonadales*, and *Anaeromyxobacter*, which was assigned to *Myxococcales*, could couple reduction of insoluble Fe(III) minerals or nitrate to oxidation of organic compounds, and they could be used in bioremediation of contaminated environments^15, 43^. Another genus *Pelobacter* was considered to have a fermentative metabolism and a narrow range of available substrates. However, *Thioalkalivibrio* of the *gamma-proteobacteria* was mixotrophic in sediments. The bacteria could not only utilize organic compounds but also H_2_S as electron donors to perform dissimilatory NO_3_^−^ reduction^44^. As shown in the heatmap (Fig. 4b), the relative abundances of different bacteria varied from the four sites in Yellow River Estuary.

From the qPCR results of different genes, denitrification was the dominant nitrate reduction process in the Yellow River Estuary, where high nitrate concentration existed. DNRA bacteria prefer a low nitrate environment and conserve ammonia in the estuary^15^. A high proportion of denitrifying bacteria contributed to the reduced nitrogen loading in the estuary. This study reveals DNRA community diversity across different sites in the Yellow River Estuary and the effects of main environmental parameters on DNRA bacteria distribution, which contribute to enrichment of the DNRA and nitrogen removal coupled with other bacteria in the laboratory.

## Conclusion

This study investigated the activity and community composition of DNRA bacteria in sediments of the Yellow River Estuary for the first time. The potential rates of DNRA bacteria varied from 0.10 to 3.29 nmol N g^−1^ h^−1^. The *nrfA* gene copy number ranged from 3.19 × 10^9^ to 2.27 × 10^10^ copies g^−1^ dry sediment. However, the activities and abundances of DNRA bacteria did not have a tendency to change along the salinity and nitrate gradients. They were more closely related to sediment organic carbon content and extractable NH_4_^+^ in sediment. The highest community diversity occurred at site 5. Based on *nrfA* gene pyrosequencing, *delta-proteobacteria* was the most abundant at all sites. This study improves our understanding of DNRA in the Yellow River Estuary. However, the contributions of anammox, denitrification and DNRA to nitrate reduction based on activity analysis require further study.

## Methods

### Study area

The Yellow River Estuary is located in Shandong province, China (Supplementary Fig. S4). It is a continental estuary with a wake tide. The estuary mouth is situated in the confluence of the Bohai Sea and the Laizhou Bay. There is a high loading of nitrogen in the estuary due to anthropogenic activity. The region has a typical temperate and monsoonal climate with four distinct seasons. In spring, due to less precipitation, rapid temperature rise, strong evaporation, spring agriculture water consumption and low estuarine water level, seawater intrusion occurred. The colour of the water turned from yellow to blue at this stage.

### Sample collection

Sediment and bottom water were collected in May, 2016 from the estuary mouth site 1 (37°48′34″N, 119°14′40″E), two mid-way sites 2 (37°47′45″N, 119°14′17″E), 3 (37°45′38″N, 119°10′18″E), one wharf site 4 (37°45′41″N, 119°9′47″E) and one flying bridge site 5 (37°45′40″N, 119°9′42″E). Surface sediments were sampled by taking 0–10 cm mixed sediments using a Peterson mud sampler and then were transported to laboratory under refrigeration for later ^15^N slurry incubation. Subsequently, some sediment samples were freeze-dried for later analysis and stored at −20 °C. Bottom water was collected using Niskin bottles and filtered through 0.45 µm syringe filters.

### Environmental parameters measurements

Environmental parameters including water column depth, temperature, DO were measured at each sampling site using a ruler and a digital portable DO metre (HQ40d, Hach, USA). pH and salinity were measured by a pH metre (PhS-3C, Rex Electric Chemical, China) and a conductivity metre (STEC-100, SP, USA), respectively, in the laboratory. The concentrations of NO_3_^−^, NO_2_^−^ and NH_4_^+^ were determined using an ultraviolet-visible spectrophotometer (TU1810-PC, Purkinje General, China) following standard methods.

After freeze drying, the sediment organic carbon content was measured with the potassium dichromate method^45^. Sediment samples were extracted for NO_3_^−^, NO_2_^−^, NH_4_^+^ analysis using 2 M KCl and then were determined as water samples^46^.

### DNRA rate

^15^N tracer slurry incubation was conducted to measure the DNRA capacity with modified method according to Mørkved *et al*.^47^. Each homogenized sediment sample was divided into four sub-samples. Approximately 33 g of sediment sample was then mixed with 30 ml of degassed (oxygen free nitrogen, OFN) synthetic river water in gas-tight vials (100 ml)^48^. Then, the vials were pre-incubated in a dark incubator (250D, Jin Yi, China) for 24 h at room temperature (20–23 °C) to deplete the residual oxygen and nitrate. After pre-incubation, ZnCl_2_ (3 ml 50% w/v) was injected into one of the four sub-samples to inhibit the microbial activity through the septum. Natural abundance ^15^N and ambient nitrate of samples were measured to use as controls. Then, the sub-samples were cultured for 6 h in a constant temperature oscillation incubator (T/C, Tai Cang, China) after injecting a degassed (OFN) stock of labelled ${}^{15}{\rm{N}}{{\rm{O}}}_{3}^{-}$ (3 ml of 2.4 mM Na${}^{15}{\rm{N}}{{\rm{O}}}_{3}^{-}$ [99.3 ^15^N atom %]) through a gas-tight syringe. The vials were opened to discharge N_2_ and then NH_4_^+^ in the sediment was extracted with 2 M KCl (4:1)^49^. The extract was filtered through a 0.45 μm PTFE syringe filter and the water samples were stored at −20 °C for later measurement of NH_4_^+^.

${{\rm{NH}}}_{4}^{+}$ diffusion was achieved using the method including the Teflon acid trap diffusion procedure according to Brooks *et al*. and Conway^50, 51^. The extract (40 ml) is placed in a specimen cup, and the Teflon Acid Trap is then placed in the cup. Then, MgO (0.2 g) was added to buffer the solution to approximate pH 10.5. The NH_3_ volatilizes and then is trapped on paper acidified with KHSO_4_. After 6 d of incubation, the paper was removed, dried, wrapped in a tin capsule, and analysed with an ANCA mass spectrometer. The DNRA rate in sediment samples was calculated according to Silver *et al*.^52^:

$${\rm{DNRA}}=\frac{(\,[{}^{{\rm{15}}}{\rm{N}}{{\rm{H}}}_{{\rm{4}}}{ \% ]}_{{\rm{f}}}-{[{}^{{\rm{15}}}{\rm{N}}{{\rm{H}}}_{{\rm{4}}} \% ]}_{{\rm{i}}})\cdot {[\mathrm{NH}}_{{\rm{4}}}]}{[{}^{{\rm{15}}}{\rm{N}}{{\rm{O}}}_{{\rm{3}}} \% ]\cdot {\rm{t}}}$$

where [^15^NH_4_%]_i_ is initial atom % of ${}^{15}{\rm{N}}{{\rm{H}}}_{4}^{+}$, [^15^NH_4_%]_f_ is the final atom % of ${}^{15}{\rm{N}}{{\rm{H}}}_{4}^{+}$, [^15^NO_3_%] is the atom% of the added nitrate tracer, the extractable ammonium concentration [NH_4_] was measured using the Nessler’s reagent Spectrophotometric method on sediment slurries (μmol g^−1^ dry soil), and “t” represents the incubation time.

### DNA extraction and qPCR of *nrfA* gene

DNA was extracted from dry sediment samples (0.65 g) using the PowerSoil DNA Kit (MO BIO Laboratories, USA) according to manufacturer’s instructions. DNA concentrations were measured on a spectrophotometer (K5500, KAIAO, China). To quantify the abundance of DNRA bacteria, qPCR assays for functional gene (*nrfA*) were performed, and the primers (*nrfA2aw*, *nrfAR1*) that target *nrfA* gene were used^40^. Each PCR mixture (20 μL) contained 10 μL of SYBR Premix Ex Taq, 0.4 μL of forward and reverse primers (5 μmol/mL), 8.2 μL dd H_2_O (TaKaRa, Japan) and 1 μL DNA or standards. The standard curve of *nrfA* gene was generated from a series of 10-fold dilutions of a plasmid DNA with an insert of *nrfA* gene in *Escherichia coli*. The PCR amplifications and quantification were performed in the LightCycler® 480 II system (Roche, Switzerland). The conditions were the same as those used by Song *et al*.^33^. The final qPCR data analysis was performed using the Abs Quant/2nd Derivative Max^53^.

### High-throughput sequence of *nrfA* gene

The DNRA community diversity of DNRA bacteria was determined on an Illumina MiSeq sequencer. Sites 1, 2, 3 and 5 were selected to examine the community structure. After DNA extraction, PCR was conducted in triplicate using PCR Amplifier 2720 under the same conditions as those of Song *et al*.^33^. The 25 μL PCR reaction system contained 5 μL of 5*Reaction Buffer, 5 μL of 5* High GC Buffer, 0.5 μL of dNTP (10 mM), 1 μL of DNA, 1 μL of each primer (10 μM), 11.25 μL of dd H_2_O (TaKaRa, Japan) and 0.25 μL of Q5 DNA polymerase (Q5™ High-Fidelity DNA Polymerase, NEB, USA). The PCR amplified product was excised from 2% agarose gels and then purified using AMPure Beads (Beckman Coulter, USA). After that, PCR result was quantified using a PicoGreen dsDNA Assay Kit (Thermo Scientific, USA) on the TBS-380 Fluorometer (Turner Biosystems, CA, USA). Then, paired-end pyrosequencing was conducted using MiSeq Reagent Kit V3 (Life Sciences, Branford, CT, USA) by the Illumina MiSeq sequence platform at the Personalbio (Shanghai, China).

### Sequence analysis and phylogenetic classification

Raw reads of *nrfA* gene sequences were saved in the FsatQ file. The bioinformatic pipeline was listed in Supplementary Fig. S5. First, the sequences shorter than 150 bp and lower than 20 quality score, as well as the sequences containing ambiguous base N, were removed through the sliding window method. Paired reads were merged based on the overlap of bases using FLASH (http://ccb.jhu.edu/software/FLASH/)^54^. Then, valid sequences were obtained through the identification between the merged reads and the relevant samples. Chimera sequences were removed using QIIME (http://qiime.org/)^55^ to get high quality sequences. Then, they were clustered into OTUs using UCLUST^56^ with 97% similarity and rare OTUs containing lower than 0.001% of total *nrfA* sequences were omitted^57^. The sequences with highest abundance of each OTU as representative sequences were annotated with NCBI taxonomy using QIIME. Microbial community richness indices included ACE and Chao1 estimations, while diversity was quantified using the Simpson and Shannon indexes. The alpha diversity index of each sample was determined using QIIME after a flattening process sequence. Rank abundance curve was drawn using R software. The length of X-axis represented richness and slope of lines represented evenness. A steep gradient indicated low evenness. The differences of community composition among four sites along the salinity gradient were evaluated through PCA based on community composition at genus level using QIIME software. Distributions of DNRA bacteria in four samples at different levels were determined using QIIME, and a heat map was constructed with the 50 most abundant genera using R software. In addition, RDA was conducted to evaluate the relationship between environmental factors and community structures using R software.

### Data processing and analysis

Pearson’s product correlation analysis was performed using SPSS statistics 17.0 package to explore the relationship among DNRA rate, *nrfA* gene abundance, the diversity index and dominant environmental parameters. Linear regression analyses between DNRA rates and *nrfA* gene abundances were conducted using Origin 9.0 and SPSS statistics 17.0.

## Electronic supplementary material

Supplementary Information
