# Supplementary material for: Dissimilatory Nitrate Reduction to Ammonium in the Yellow River Estuary: Rates, Abundance, and Community Diversity
Source: Sci Rep. 2017 Jul 28;7:6830. doi: 10.1038/s41598-017-06404-8 (PMC5533712; doi:10.1038/s41598-017-06404-8)

Supplementary Information

Dissimilatory Nitrate Reduction to Ammonium in the Yellow River Estuary: Rates, Abundance, and Community Diversity

Cuina Bu, Yu Wang, Chenghao Ge, Hafiz Adeel Ahmad, Baoyu Gao, Shou-Qing Ni *

Shandong Provincial Key Laboratory of Water Pollution Control and Resource Reuse, School of Environmental Science and Engineering, Shandong University, Jinan 250100, China

*Corresponding author：Shou-Qing Ni, School of Environmental Science and Engineering, Shandong University, Jinan, Shandong, PR China, 250100. E-mail: sqni@sdu.edu.cn.

Supplementary Figures

Legends for Supplementary Figure

Supplementary Figure 1. Venn diagram of OTUs in the four sites of the Yellow River Estuary.

Supplementary Figure 2. Rank abundance curve based on *nrfA* sequencing in the Yellow River Estuary.

Supplementary Figure 3. Functional gene abundance of two dissimilatory nitrate reduction processes (DNRA and Denitrification) in the Yellow River Estuary. Genes include *nirS* (cytochrome cd1 nitrite reductase), *nirK* (copper containing nitrite reductase), *nosZ* (nitrous oxide reductase) and *nrfA* (periplasmic nitrite reductase). Error bars represent standard deviation.

Supplementary Figure 4. Sampling sites in the Yellow River Estuary, Shandong, China. The figure was generated according to the distribution of land, sea and river using Adobe Photoshop CS5 (http://www.52z.com/soft/23642.html).

Supplementary Figure 5. Bioinformatic pipeline of *nrfA* pyrosequencing analysis.

Supplementary Table 1. Environmental parameters of bottom water and sediment in the five sites of the Yellow River Estuary.

Study site		1(mouth)	2	3	4	5
Bottom water	Depth (m)	1.3	2	4	3	5
	Temperature (℃)	25.0	23.4	21.0	20.8	20.6
	Salinity (‰)	22.1	21.3	7.7	5.1	4.2
	pH	7.94	7.94	8.08	8.03	8.05
	DO (mg/L)	8.92	9.19	10.31	9.90	9.76
	NO_3^- (μM)	102.86	159.29	229.29	269.29	272.14
	NH_4^+ (μM)	155.71	118.57	110.71	110.71	118.57
Sediment	Moisture content (%)	20.76	23.62	28.22	22.07	45.11
	Organic carbon (g/kg)	3.60	4.28	5.37	3.95	6.71
	Extractable NH_4^+ (μmol/g)	0.28	0.10	0.40	0.18	0.81

Supplementary Table 2. DO saturation in the five sites of the Yellow River Estuary.

Study site	Temperature ℃	DO mg/L	DO saturation %
1	25.0	8.92	108
2	23.4	9.19	107
3	21.0	10.31	116
4	20.8	9.90	111
5	20.6	9.96	112

Supplementary Table 3. DNRA rate and *nrfA* gene abundance in the five sampling sites of the Yellow River Estuary. ds: dry sediment.

Study site	DNRA rate nmoles N g^{-1} h^{-1}	nrfA gene copies g^{-1} ds
1	0.19	$3.19 \times 10^{9} \pm 1.38 \times 10^{8}$
2	0.10	$6.00 \times 10^{9} \pm 5.81 \times 10^{8}$
3	1.52	$2.27 \times 10^{10} \pm 1.75 \times 10^{9}$
4	0.21	$8.65 \times 10^{9} \pm 1.61 \times 10^{8}$
5	3.29	$1.74 \times 10^{10} \pm 9.27 \times 10^{7}$

Supplementary Table 4. Number of *nrfA* sequences, richness and diversity in the Yellow River Estuary.

Study site	Valid reads	High quality reads	OTUs	Chao 1	ACE	Simpson	Shannon
1	114,138	104,395	4,511	3,680	3680.00	0.9907	9.34
2	169,444	145,453	5,699	4,512	5256.75	0.9906	9.04
3	205,444	174,960	6,022	5,068	6316.29	0.9895	9.12
5	205,114	165,790	6,093	5,240	6444.56	0.9926	9.36

Supplementary Table 5. OTUs classification at different levels.

Study site	Phylum	Class	Order	Family	Genus	Species
1	21	47	55	77	106	121
2	25	55	68	86	114	128
3	25	58	65	78	102	116
5	23	54	61	72	95	107

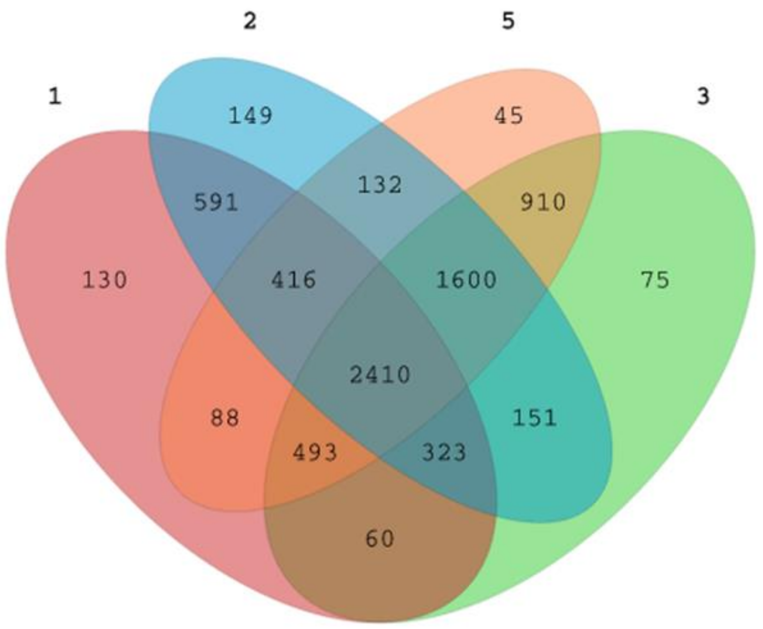

Supplementary Figure 1. Venn diagram of OTUs in the four sites of the Yellow River Estuary.

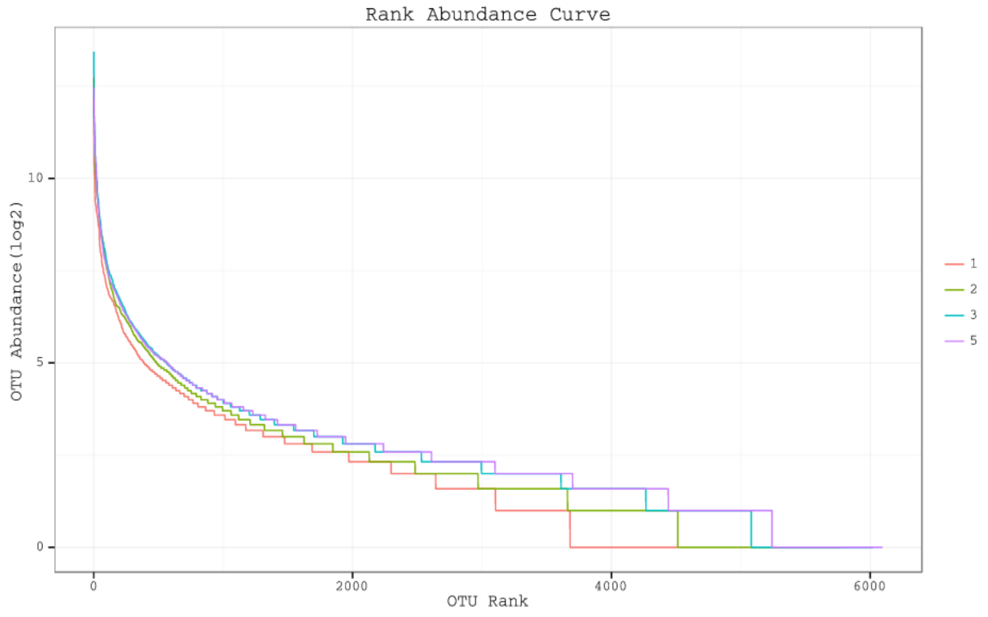

Supplementary Figure 2. Rank abundance curve based on *nrfA* sequencing in the Yellow River Estuary.

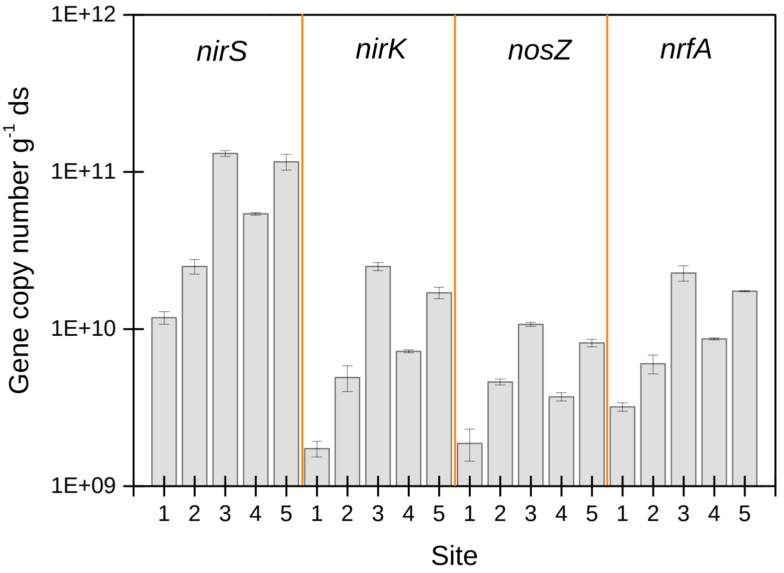

Supplementary Figure 3. Functional gene abundance of two dissimilatory nitrate reduction processes (DNRA and Denitrification) in the Yellow River Estuary. Genes include *nirS* (cytochrome cd1 nitrite reductase), *nirK* (copper containing nitrite reductase), *nosZ* (nitrous oxide reductase) and *nrfA* (periplasmic nitrite reductase). Error bars represent standard deviation.

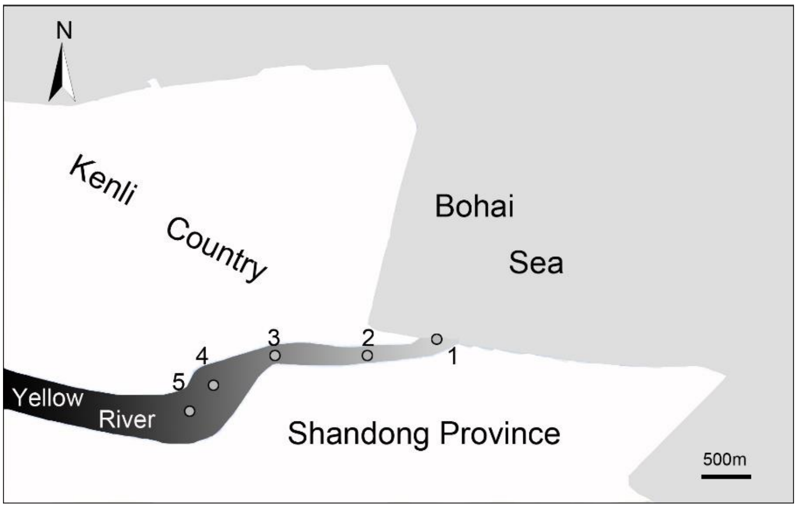

Supplementary Figure 4. Sampling sites in the Yellow River Estuary, Shandong, China. The figure was generated according to the distribution of land, sea and river using Adobe Photoshop CS5 (http://www.52z.com/soft/23642.html).

Supplementary Figure 5. Bioinformatic pipeline of *nrfA* pyrosequencing analysis

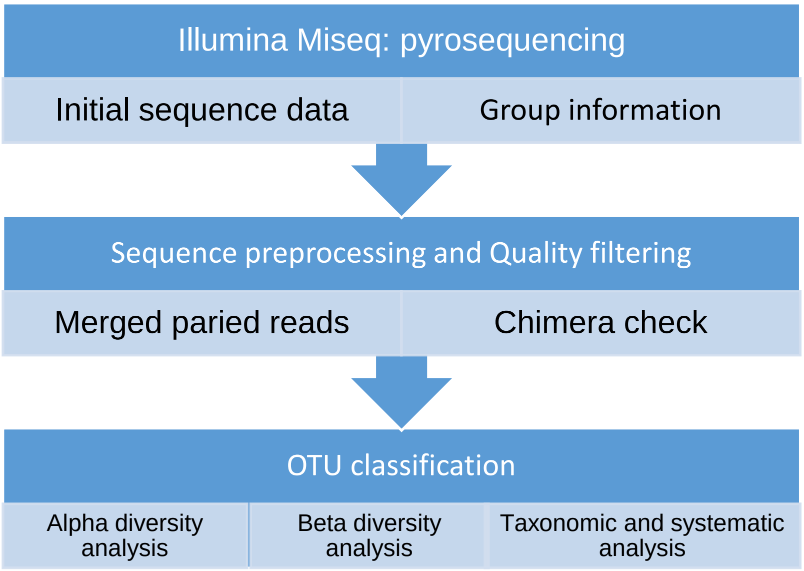

Supplement: Supplementary file 1 — Supplementary Information [file 41598_2017_6404_MOESM1_ESM.pdf]
